# Sweepers in the CNS: Microglial Migration and Phagocytosis in the Alzheimer Disease Pathogenesis

**DOI:** 10.1155/2012/891087

**Published:** 2012-05-14

**Authors:** Mariko Noda, Akio Suzumura

**Affiliations:** Department of Neuroimmunology, Research Institute of Environmental Medicine, Nagoya University, Furo-cho, Chikusa-ku, Nagoya 464-8601, Japan

## Abstract

Microglia are multifunctional immune cells in the central nervous system (CNS). In the neurodegenerative diseases such as Alzheimer's disease (AD), accumulation of glial cells, gliosis, occurs in the lesions. The role of accumulated microglia in the pathophysiology of AD is still controversial. When neuronal damage occurs, microglia exert diversified functions, including migration, phagocytosis, and production of various cytokines and chemokines. Among these, microglial phagocytosis of unwanted neuronal debris is critical to maintain the healthy neuronal networks. Microglia express many surface receptors implicated in phagocytosis. It has been suggested that the lack of microglial phagocytosis worsens pathology of AD and induces memory impairment. The present paper summarizes recent evidences on implication of microglial chemotaxis and phagocytosis in AD pathology and discusses the mechanisms related to chemotaxis toward injured neurons and phagocytosis of unnecessary debris.

## 1. Introduction

Microglia are macrophage-like resident immune cells in the central nervous system (CNS) and possess both neurotoxic and neuroprotective function. Microglia accumulate in the lesions of a variety of neurodegenerative disorders, such as Alzheimer's disease (AD), Parkinson's disease, and multiple sclerosis, and are thought to play both toxic and protective functions for neuronal survival [1]. Microglia are considered to be a first line defense and respond quickly to various stimuli. When activated, microglia undergo morphological changes to ameboid, proliferate, migrate toward injured areas, and release many soluble factors and phagocytosis of foreign substances or unwanted self-debris. Appropriate migration of microglia to damaged area is controlled by chemokines and nucleotide ATP [2, 3]. Phagocytosis seems to be important to prevent the senile plaque expansion in AD by removing amyloid *β* (A*β*) deposit [4]. Microglia not only engulf the A*β* protein but also phagocytose apoptotic cells and degenerated neuronal debris. Phagocytosis of apoptotic or degenerated neuronal debris is crucial to reduce inflammation and maintain healthy neuronal networks. Another type of phagocytosis, phagocytosis with inflammation, occurs in chronic inflammatory-related neurodegenerative disorders including Alzheimer disease [5–7].

Degenerated neurons releases several signaling molecules, including nucleotides, cytokines, and chemokines, to recruit microglia and enhance their activities [8, 9]. The phenomenon are now termed as find-me, eat-me, and help-me signals. In this paper, we focused on find-me, and eat-me signals from degenerated neurons to microglia. Most distinguished and examined eat-me signal is phosphatidylserine (PS), which is a component of cellular membrane and is everted on apoptotic cell membrane [10, 11]. Nucleotides are also considered as the eat-me signal lately; microglia expresse various P2X and P2Y receptors, nucleotide receptors, which regulate not only chemotaxis but also phagocytosis [8, 12].

**Figure 1:**
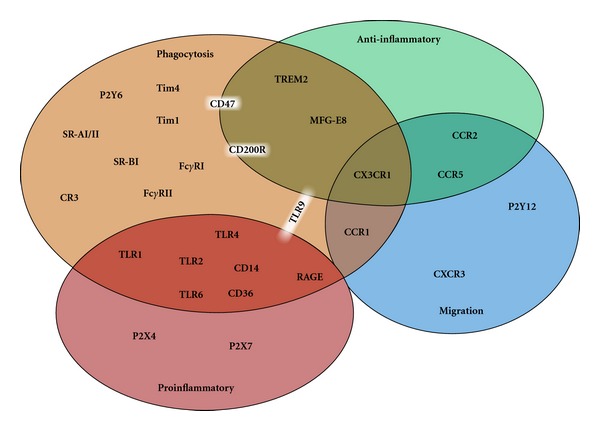
Chemotaxis or phagocytosis-involved receptors in microglia and correlation of the inflammatory or anti-inflammatory response. Many of the receptors correlated with microglial activities of chemotaxis (migration) or phagocytosis, respectively. Among these, some of the receptors possess not merely single function; CCR1 is the migration-inducing receptor that also possesses phagocytotic activity. CCR2 and CCR5 are also the migration-inducting receptors that lead to anti-inflammatory response. CX3CR1 contributes to migration, phagocytosis, and anti-inflammatory response. TREM2 and MFG-E8 induce phagocytosis and anti-inflammatory response. CD47 and CD200R usually induce phagocytosis under pathological condition, so that they indirectly contribute to anti-inflammatory status. TLR9 activates microglia to induce phagocytosis with producing proinflammatory and anti-inflammatory molecules. There are receptors inducing not only phagocytosis but also inflammatory response (CD14, CD36, RAGE, TLR1, TLR2, TLR4, and TLR6). Within these receptors that the synergistic signaling involved A*β*-triggering inflammatory response are CD14-TLR2-TLR4 and CD36-TLR2-TLR6.

**Figure 2:**
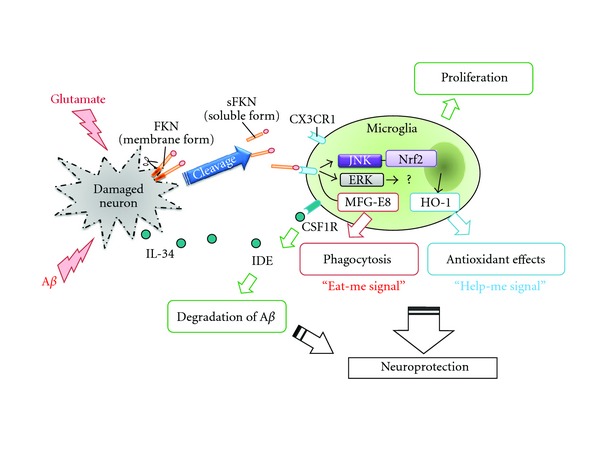
Model of the role of neuronal chemokine (FKN) and neuronal cytokine (IL-34) in microglial phagocytosis and neuroprotection. Neuronal cells primary produce chemokine fractalkine (CX3CL1; FKN) and cytokine IL-34. Microglia predominantly express its receptor, CX3CR1, and colony-stimulating factor 1 receptor (CSF1R). Soluble form of FKN (sFKN) is secreted from damaged neurons and promotes microglial phagocytosis of neuronal debris through the release of MFG-E8. sFKN also induces the expression of the antioxidant enzyme HO-1 in microglia via Nrf2 recruitment and activation of the JNK MAPK signaling pathway. The neuroprotective effects of sFKN are also mediated in part by activation of ERK MAPK, although the downstream signaling pathway has not yet been elucidated. IL-34 promoted microglial proliferation and clearance of A*β* which mediates insulin-degrading enzyme (IDE) expression. Therefore, sFKN and IL-34 may be an intrinsic neuroprotectant for damaged yet surviving neurons.

Microglia express many other surface receptors, which have direct interaction with the target to initiate phagocytosis, including PS receptor [6], lipopolysaccharide (LPS) receptor CD14 [13], the scavenger receptor CD36 [14], the purine receptor P2Y6 [8], and the toll-like receptors (TLRs) [15] (Table 1, Figure 1). Another surface receptor, the CX3C chemokine fractalkine receptor CX3CR1, is almost exclusively expressed in microglia throughout the CNS, which is involved in progression of neurodegenerative disease by altering microglial activities [16, 17] (Figure 2). Deletion of CX3CR1 expression in microglia results in progressive neuronal cell death in an animal model of neurodegenerative disease, by inducing microglial dysfunction. It has been identified recently that neurons themselves produce cytokine and chemokine, such as fractalkine. As shown in Figure 2, we previously reported that interleukin-34 (IL-34), a newly discovered cytokine, is produced by neurons, and that its receptor, colony-stimulating factor 1 receptor, is primarily expressed on microglia [18]. Fractalkine and IL-34 might be important mediator between neurons and microglia, and it is important to clarify this cellular crosstalk signaling pathways for seeking future therapeutic target of neurodegenerative diseases including AD. In the following sections, we will discuss about recent advances of microglial chemotaxis and phagocytosis and their implications for AD therapy.

## 2. Microglial Chemotaxis in CNS Injury

Chemokine and its receptors are expressed in broad area of the CNS. The expression levels were increased under pathological conditions, which seem to facilitate the recruitment and trafficking of glial cells to the damaged area [22]. Microglia constitutively express several chemokine receptors (Table 1), which are implicated in the recruitment and accumulation microglia in AD lesions. When exposed to A*β*, microglia are induced to produce several chemokines, such as CCL2, CCL4, and CXCL12 [23]. CCR2, the receptor of CCL2, deficiency resulted in reduction of microglial accumulation and higher brain A*β* levels in mouse model of AD, which might be mediated via suppression of anti-inflammatory molecule, transforming growth factor *β* (TGF-*β*) [3]. However, there is a conflicting report showing increased TGF-*β* signaling in microglia surrounding A*β* plaques in CCR2 knockout in AD model mouse (APP_Swe_/PS1/CCR2^−^/^−^) [24]. The other chemokine receptor CX3CR1 expression in microglia was also increased in the mice. This AD model has been shown to have accumulation of oligomeric A*β* and memory impairment [24]. CX3CR1 is the sole receptor of CX3C chemokine CX3CL1 (fractalkine). The roles of CX3CL1-CX3CR1 signaling on AD pathology are discussed in next section. CCL2 expression level is also related to another neurodegenerative disorder, multiple sclerosis (MS); CCL2 level is downregulated in cerebrospinal fluid from MS patients [25].

CCL21 is a neuronal chemokine, expressed in neurons. Expression of CCL21 is upregulated in neurons undergoing degeneration [26, 27]. CCL21 triggers chemotaxis of microglia through CXCR3, but not CCR7 which implicated in peripheral lymphoid organs [28].

CXCR3-CXCL10 interaction is also implicated in microglial migration [29]. CXCR3 knockout mice reveal impairment of the microglial migration but no change in proliferation. CXCL10 is also expressed in neurons. CXCL10 and CCL21 synergistically induce microglial homing through the receptor CXCR3 [30]. CXCL10 inhibits CCL21-induced migration in microglia through CXCR3 [31]. Neuronal CCL21 upregulates P2X4 receptor, the nucleotide receptor, expressed in microglia [32]. This cascade is implicated in pathophysiology of tactile allodynia to cause chronic neuropathic pain.

CCR5 also plays a role in neuronal survival [33]. In ischemic stroke models, brain damage is severer by CCR5 deficiency [34].

Inter- and intracellular transmitter nucleotides can influence microglial migration and phagocytosis. Microglia express various nucleotide receptors, P2X and P2Y receptors (Table 1) [35]. A*β*-induced microglial activation is mediated through P2X7 receptor that is reported as proinflammatory response conductive receptor [36]. ATP predominantly induced microglial migration among nucleotides through P2Y receptors, especially P2Y12 [2, 37]. Following CNS injury, expression of P2Y12 in microglia drastically reduced after microglial activation, suggesting that P2Y12 is a primary and temporary receptor to induce microglial chemotaxis at early stages of the local CNS injury [37]. The other nucleotide UDP increases mainly microglial phagocytosis (uptake of microspheres) via P2Y6 receptor [8]. In the condition brain damage by kainic acid administration, the P2Y6 receptor is upregulated and can act as a sensor for phagocytosis [38].

## 3. CX3CL1-CX3CR1

The CX3C chemokine CX3CL1 (fractalkine, also called as neurotactin), which has been identified as two forms, soluble or membrane-anchored forms, plays a pivotal role in signaling between degenerating neurons and microglia [39]. CX3CL1 and its receptor CX3CR1 are highly expressed in brain tissue, particularly in neurons and microglia [40–42]. CX3CL1 directly induces various microglial functions including migration [41], proliferation [43], inhibition of Fas-ligand-induced cell death [44] and glutamate-induced neurotoxicity [45, 46], and inhibition of proinflammatory cytokine production [42, 47]. Recently, we have shown that soluble form of CX3CL1 also directly enhances microglial clearance of degenerated neuronal debris, which is mediated through phosphatidylserine (PS) receptor and production of Milk fat globule-EGF factor 8 protein (MFG-E8) [46] (Figure 2). The source of soluble form of CX3CL1 is neuron. The membrane-anchored CX3CL1 is cleaved by several proteases including a disintegrin and metalloprotease (ADAM) family, ADAM-10 and ADAM-17 [48–50], and cathepsin S [51]. When neurons are injured or exposed to glutamate, shedding of CX3CL1 occurs immediately [41, 52]. However, little is known about direct connection with A*β*-induced neuronal toxicity and the CX3CL1-shedding.

Another important function of ADAM family enzyme, except for CX3CL1 shedding, is an *α*-secretase. It cleaves APP in the centre of the A*β* domain, and generated *α*-APP is considered to have neurotrophic function [53–56]. The ADAMs include ADAM-9, ADAM-10, and ADAM-17 [57, 58]. Cathepsin S is expressed predominantly in microglia and implicated in microglial activation of neuropathic pain [51]. Therefore, these CX3CL1-shedding proteases may regulate the microglial phagocytosis directly or indirectly through CX3CL1 expression, and may also play a role on pathogenesis of AD.

Microglia respond to CX3CL1 through CX3CR1. In a previous study, we have shown that CX3CL1 functions neuroprotective against activated microglia-induced neurotoxicity [46]. There are some reports showing that CCL2 activates CX3CR1 expression, and the induction of CX3CR1 expression by CCL2-CCR2 axis is mediated through p38 MAPK activation [59]. CX3CR1 deficiency increases susceptibility to neurotoxicity in mouse models of Parkinson's disease, amyotrophic lateral sclerosis, and systemic LPS administration [16]. CX3CL1-induced neuroprotection in a rat model of Parkinson's disease has also been reported recently [60]. In addition, there are some reports showing that pathologic features of AD mouse model are worsened by knockout of CX3CR1 [17, 61].

## 4. Microglial Receptors Involving Phagocytosis with or without Inflammation: Possible Implication to AD Pathology

Microglial phagocytosis is classified into two categories, with or without inflammation [62]. Some receptors are involved in both types of phagocytosis (Table 1). The receptor leading to inflammatory status includes CD14, CD36, the receptor for advanced glycation end products (RAGEs), and toll-like receptor (TLR) 1, TLR2, TLR4, and TLR6. The receptor leading to anti-inflammatory status includes triggering receptor expressed on myeloid cells 2 (TREM2) and PS receptor (PSR), MFG-E8. Microglia also express many other phagocytosis-related receptors which are not yet unclear to the correlation of inflammatory status.

**CR3**

Microglia express classical phagocytosis-related receptor, the *α*_M_ *β*_2_ integrin complement-receptor-3 (CR3; MAC-1; CD11b/CD18), and scavenger-receptor (SR)-AI/II (SR-AI/II; CD204). CR3 synergistically activated SR-AI/II-mediated myelin phagocytosis by microglia [63, 64]. CR3 is implicated in clearance of bacteria through induction of major histocompatibility complex class II (MHC II) antigens [65]. CR3 is involved in endocytosis in normal conditions and MHC II antigens in an inflammatory state. Microglia express SR-AI/II and SR-BI during neonatal period, while in adult they lack the expression. However SR-AI/II expression in microglia is upregulated in AD [66].

**CD14**

CD14 is the LPS receptor which is also considered as classical phagocytosis-related receptor in macrophage and microglia [67]. CD14-mediated phagocytosis of apoptotic cells occurred in both normal and inflammatory conditions [67]. CD14-mediated phagocytosis does not require expression of PS receptor and possibly induces inflammatory conditions through activation of CD14 signaling [68]. CD14 also contributes phagocytosis of A*β* by microglia [13]. However, deletion of CD14 reportedly attenuates pathological features of AD mouse model. The authors suggested that it might be due to reduction of insoluble, but not soluble, A*β* [69].

**Fc*γ*Receptor**

Exposure of fibrillar A*β* to microglia induces phagocytosis through the receptors distinct from those used by the classical phagocytosis: immunoglobulin receptors (Fc*γ*RI and Fc*γ*RIII) or complement receptors [70].

**CD36 and CD47**

A*β* directly interacted with microglial cell surface receptor complex comprising the class B scavenger receptor CD36, *α*_6_ *β*_1_ integrin, and integrin-associated protein CD47, all of them involved in migration and phagocytosis of microglia [70–72]. CD36 is required for fibrillar A*β*-induced chemotaxis and proinflammatory molecules including reactive oxygen species (ROS), TNF*α*, IL-1*β*, and several chemokines in microglia [23]. A*β* activates microglial recruitment to amyloid deposition site through CD36-dependent signaling cascade involving the Src kinase family members, Lyn and Fyn, and the ERK1/2 [73]. CD47 is a membrane glycoprotein, broadly expressed in the various cell types in the CNS, including neurons and microglia. Signal regulatory protein-*α* (SIRP*α*; CD172a) is a receptor binding to CD47, which is also expressed on neurons and myeloid cells. SIRP*α* is an inhibitory molecule of CD47 that downregulates MAPK phosphorylation which is a downstream pathway of CD47 [74]. SIRP*α* interacts with the protein tyrosine phosphatases SHP-1 and SHP-2, which are predominantly expressed in neurons, dendritic cells, and macrophages [75]. Intact myelin expresses CD47 to suppress myelin phagocytosis by microglia via SIRP*α*-CD47 interaction [76]. Therefore, CD47 functions normally as a marker of “self” to protect intact body component [76, 77].

**RAGE**

In has been reported that the direct interaction of A*β* peptide with the receptor for RAGE is important in AD pathophysiology. In AD brains, RAGE expression is increased, and RAGE directly induces neurotoxicity by production of oxidative stressors and indirectly by activating microglia [78]. RAGE increases macrophage-colony stimulating factor (M-CSF) from neurons via nuclear-factor-*κ*B- (NF-*κ*B-) dependent pathway and released M-CSF induced interaction of cognate receptor c-fms on microglia which prolongs survival of microglia [79]. RAGE recognizes multiple ligands other than A*β* peptide, such as advanced glycation end products (AGEs), PS, and high-mobility group box 1 protein (HMGB1) [80, 81]. These molecules act as an agonist of RAGE on microglia, by inducing proinflammatory molecules, such as NO, TNF-*α*, IL-1*β*, and IL-6, via MAP-kinase-kinase (MEK) and phosphatidylinositol 3-kinase (PI3K) pathways [81]. Activation of RAGE leaded to NF-*κ*B and MAPK-mediated signaling to propagate and perpetuate inflammation status [82]. RAGE also mediates the transport of peripheral A*β* into the brain across the blood-brain barrier (BBB) [83].

**CD200R**

CD200 is a transmembrane glycoprotein and is expressed on many different cell types including neurons, endothelial cells, lymphocytes, and dendritic cells [84]. The receptor of CD200, CD200R expression, is predominant in myeloid cells, macrophages, and microglia [84, 85]. As in the case of SIRP*α*-CD47, CD200 exerts inhibitory effect on CD200R, so that CD200-CD200R interaction can downregulate activity of microglia. In retina, it has been shown that activation of CD200R in microglia does not show direct effect on migration, but CD200-CD200R signaling restores LPS/IFN*γ*-induced loss of migration [86]. CD200 knockout leads to an expansion of the myeloid population in several tissues and increased expression of the activation markers in microglia, including the signaling adaptor protein DNAX-activating protein of 12 kDa (DAP12), CD11b, CD45, CD68, and inducible NO synthase (iNOS) [87]. Blocking of CD200R increases neurodegeneration in mouse model of Parkinson's disease [88]. These observations suggest that CD200-CD200R signaling leads to anti-inflammatory state and protection against neurotoxic stimuli. CD200 and CD200R expression levels (neurons and microglia, resp.) are decreased in AD hippocampus and inferior temporal gyrus, indicating that inhibition of CD200-CD200R axis contributes to AD pathology [89].

**TREM2**

TREM2, a recently identified innate immune receptor, and its adaptor protein DAP12 are expressed on microglia and cortical neurons, but not on hippocampal neurons, astrocytes, and oligodendrocytes. Their expression correlates with clearance of apoptotic neurons by microglia without inflammation [90–92]. However, endogenous ligand or specific agonist of TREM2 had not been identified until recently. Heat shock protein 60 (Hsp60) is a mitochondrial chaperone, which interacts with TREM2 directly. Hsp60-induced phagocytosis is only found in microglia which have robust expression of TREM2 [93]. In AD model mouse, TREM2 expression was highest in the outer zone in A*β* plaques, and the expression level correlated with the size of A*β* plaque [94]. Forced expression of TREM2 positively regulated microglial phagocytosis, the ability of microglia to stimulate CD4^+^ T-cell proliferation, TNF-*α* and CCL2 production, but not IFN*γ* production [94].

## 5. TLRs

TLRs are class of pattern-recognition receptors in the innate immune system to induce inflammatory responses. 13 TLRs have been identified in human and mouse to date, except for TLR10 which is expressed only in human [95, 96]. TLRs may also contribute to the microglial inflammatory response to promote AD pathogenesis [15].

CD11b, a marker of macrophages and microglia, has been shown to interact with TLR signaling. CD11b knockout mouse exhibited enhanced TLR-mediated responses and subsequent more susceptibility to endotoxin shock [97].

Among the TLRs, LPS receptor TLR4 potently activates microglia in various aspects, such as A*β* phagocytosis and proinflammatory molecules production [98, 99]. Activation of TLR1, TLR2, TLR3, and TLR9 by each selective agonist also increased phagocytosis and several cytokines and chemokine production [98, 100]. CD14 is a coreceptor of TLR4. The response of microglia to fibrillar A*β* is mediated via CD14, which act together with TLR4 and TLR2 to bind fibrillar A*β* and induced microglial activation through p38 MAPK [101]. Deficiency of CD200 induces the expression of TLR2 and TLR4 in glial cells and proliferation of CD11b^+^/MHCII^+^/CD40^+^ activated microglia [102]. These mice show memory impairment, suggesting that dual activation of TLR2 and TLR4 may induce an inflammatory phenotype of microglia which negatively regulate synaptic plasticity in the AD model. A*β* triggers the inflammatory status in microglia via heterodimer of TLR4 and TLR6, which is regulated by CD36 [103]. Therefore, CD14-TLR2-TLR4 and CD36-TLR4-TLR6 signaling are crucial to A*β*-induced inflammatory response, and also in microglial phagocytosis.

Bacterial DNA containing motifs of unmethylated CpG dinucleotides (CpG-DNA) is a ligand of TLR9, which is initially identified to activate microglia and strongly induces TNF-*α* and IL-12 production [104]. However, we have shown that CpG activated microglia to produce neuroprotective molecule, such as hemeoxygenase-1 (HO-1) and matrix metalloproteinase 9 (MMP-9) without producing neurotoxic molecules, such as TNF-*α*, glutamate and nitric oxide (NO), and enhanced A*β* clearance to protect memory disturbance *in vivo* [105]. There are the discrepancies about CpG effect between aforementioned two reports despite using the same origin cells (mouse primary microglia). It may be due to concentrations of this TLR9 ligand used: higher concentration (10 *μ*M) in former report [104], whereas lower concentrations (1 to 100 nM) in latter report [105]. Moreover, the latter report revealed the difference on neuroprotective effect of CpG among synthetic oligodeoxynucleotides (ODNs) classes (A to C). Class A CpG did not activate microglia, but classes B and C CpGs increased microglial neuroprotective effect through induction of clearance of A*β* and production of neuroprotectant. These suggested that the CpG sequence-dependent microglial activation and responses are present.

CpG increased chemokine CCL9 and its receptor CCR1 expression in macrophages and microglia via TLR9/MyD88 signaling involving ERK, p38 MAPK, and PI3K pathways. Thus it can enhance microglial migration as well as phagocytosis [106].

## 6. PSR

Phagocytotic cells recognize apoptotic cells by several mechanisms, including recognition of PS expressed on the cells. PS is receiving much attention because it is responsible for phagocytosis without inducing inflammation [10]. The receptors of PS (PSRs) had not been clarified for a long time but have uncovered in recent years. These include MFG-E8 (the lactadherin homolog in humans) and T-cell immunoglobulin mucin domain 4 (Tim4) [107, 108]. These act as a bridge between PS-expressing apoptotic cells and PSR expressing phagocytes and trigger engulfment of cellular debris.

MFG-E8 is expressed on various macrophage subsets in the periphery and on microglia in the CNS. Recently, we have shown that CX3CL1 induces MFG-E8 expression in primary mouse microglia to lead to the microglial clearance of degenerated neuronal debris [46]. Others also reported the induction of MFG-E8 by CX3CL1 in macrophages and rat microglia [109, 110]. MFG-E8 bridges PS and integrins *α*_v_ *β*_3_ or *α*_v_ *β*_5_ on the surface of phagocytes [107, 111]. High-mobility group box 1 protein (HMGB1) is an intracellular protein that activates transcriptional factors, including p53 and NF-*κ*B. HMGB1 reportedly suppresses the interaction between MFG-E8 and *α*_v_ *β*_3_ integrin in macrophage and inhibits the phagocytosis of apoptotic cells through ERK-mediated signaling pathway [112]. MFG-E8 may also be involved in A*β* phagocytosis, since its expression is reduced in AD [113]. We previously showed the neutralization of MFG-E8 progressed neuronal degeneration [46]. MFG-E8 reportedly induces anti-inflammation status in the periphery. Therefore, MFG-E8 may possibly lead to targeted clearance of unwanted molecules, such as A*β*, without inflammation.

The other well-studied PSR, Tim4, is expressed in MAC-1^+^ cells in various mouse tissues, including spleen, lymph nodes, and fetal liver [108]. Among the other Tim family members, Tim1, but neither Tim2 nor Tim3, also specifically binds to PS. Tim1-PS subsequently connects with exosomes to recognize and engulf apoptotic cells [114–116].

It has been shown recently that RAGE also recognizes PS and induces apoptotic cell clearance [80]. However as mentioned previously, RAGE-guided intracellular signaling pathway induces prolonged inflammatory status.

## 7. *γ*-Secretase and Phagocytosis in AD Pathology

*γ*-secretase is a protein complex of four essential membrane proteins: aph-1, pen-2, nicastrin, and presenilin. A recent study suggests that presenilin increases microglial phagocytosis of A*β*, and this *γ*-secretase has dual role for AD pathogenesis: one is cleavage of amyloid precursor protein (APP) to produce pathologic A*β*, and the other is reduction of microglial phagocytosis of A*β* by *γ*-secretase inhibitor [117]. Vertebrates have two presenilin genes, PSEN1 (located on chromosome 14 in humans; encoded presenilin 1) and PSEN2 (located on chromosome 1; encoded presenilin 2). There is a report that presenilin 2 is identified as the predominant *γ*-secretase in mouse microglia (but not presenilin 1), which repressed microglial activation via its function as a *γ*-secretase, and its expression is increased by inflammatory stimuli (IFN-*γ*) [118].

In order to explore the detailed mechanism how *γ*-secretase regulates microglial activity, further studies are needed since *γ*-secretase is a therapeutic target for AD. In traumatic injury of the brain, presenilin and nicastrin expressions are elevated in activated microglia and astrocytes [119]. *γ*-secretase mainly cleaves APP to lead to accumulation of A*β*_1-42_, which then results in aggregation of A*β* protein to worsen AD pathology. However A*β*_1-42_ is also a target of microglial phagocytosis.

## 8. Concluding Remarks

Microglia express a wide array of receptors characteristic to immune cell, such as CD molecules, integrins, chemokine receptors, and PSR (Figure 1). These receptors are involved in multiple functions of microglia. Chemokine receptors not only induce migration of microglia but also contribute directly to AD pathogenesis through regulation of phagocytosis and neuroprotective activity. PS acts as eat-me signal, and PSR-mediated phagocytosis so far is regarded as inducing anti-inflammatory responses. However, according to some recent reports, RAGE interacts with PSR and facilitates phagocytosis with robust inflammation status [80, 120]. Therefore, if the other phagocytosis-related receptors including TLRs interact with PSR, microglia would be activated to outbreak excessive phagocytosis with robust inflammation.

Microglia from old APP/PS1 mouse, but not from younger ones, show the reduction of SRA, CD36, RAGE, and the A*β*-degrading enzymes including insulysin, neprilysin, and MMP9 [121]. Dysfunction of microglia after progression of disease development may lead to neurodegeneration. Thus, it is important to consider the microglial status depending on the disease stage, to treat AD effectively. As shown in Figure 2, FKN and IL-34 may be an intrinsic neuroprotectant for damaged but still surviving neurons through activation of microglia. Therefore, it is important to elucidate neurons-microglia crosstalk in neurodegenerative condition.

## Figures and Tables

**Table 1:** Various chemotaxis or phagocytosis-related receptors in microglia and its ligand(s) or interacted factors. Microglia are activated with various stimuli through the specific receptor of each stimuli. For detailed review of chemokines, pathogens, and factors associated with tissue damage recognized by microglia, refer to [19–21].

Receptor type	Subtypes	Ligand(s)/interacted factors
	CCR1	CCL3 (MIP-1*α*), CCL5 (RANTES), CCL7 (MCP-3), CCL9 (MIP-1*γ*), CCL14 (HCC-1), CCL15 (HCC-2/leukotactin-1), CCL16 (HCC-4/LEC), CCL23 (MPIF-1)
Chemokine receptor	CCR2	CCL2 (MCP-1), CCL7 (MCP-3), CCL8 (MCP-2), CCL13 (MCP-4), CCL16 (HCC-4/LEC)
	CCR5	CCL3 (MIP-1*α*), CCL4 (MIP-1*β*), CCL5 (RANTES)
	CXCR3	CXCL9 (Mig), CXCL10 (IP-10), CXCL11 (I-TAC)
	CX3CR1	CX3CL1 (Fractalkine)

Purinergic receptor	P2X4, P2Y7, P2Y12 P2Y6	ATP, ADP UDP

TLR	TLR1 TLR2 TLR4 TLR6 TLR9	Triacyl lipopeptides Glycolipids, Hsp70, HMGB1, A*β* LPS, Hsp Diacyl lipopeptides CpG-DNA

Phosphatidylserine (PS) receptor	MFG-E8, Tim1, Tim4 RAGE	PS of apoptotic cells A*β*, AGE, HMGB1 PS of apoptotic cells

Scavenger receptor (SR)	SR-AI/II (CD204), SR-BI, CD36	Cellular debris, apoptotic cells, A*β* (CD36)

Immunoglobulin (Ig) receptor	Fc*γ*RI, Fc*γ*RIII	Ig-opsonized particles

Complement receptor (CR)	CR3 (MAC-1; CD11b/CD18)	Complement components, opsonized particles

Other phagocytosis-related receptor	CD14 CD47 CD200R TREM2	LPS, A*β* SIRP*α* (CD172a) CD200 Hsp60, DAP12
